# Supplementary material for: Co-infection of HIV or HCV among HBsAg positive delivering mothers and its associated factors in governmental hospitals in Addis Ababa, Ethiopia: A cross-sectional study
Source: PLoS One. 2022 Aug 26;17(8):e0273300. doi: 10.1371/journal.pone.0273300 (PMC9417033; doi:10.1371/journal.pone.0273300)
Supplement: S1 File — (DOCX) [file pone.0273300.s001.docx]

**Questioner on Socio demographic profiles of the study participants**

**Name of health facility _______________**

| s.no | Variables | Response |
| --- | --- | --- |
| 1 | Age (years) | ------------------------------- |
| 2 | Marital status | 1. Married 2. Single 3. Divorced 4. Widowed 5. Others specify____________________ |
| 3 | Level of education | 1. Illiterate 2. Primary level(1-8) 3. Secondary level(9-12) 4. college diploma and above |
| 4 | Current occupation | 1. Government employee  2. Private employee  3. Non-government organization employee  4. Self-employee  5. Nil  6. House wife  7. Daily laborer  8. Others (specify)________________________ |
|  | Religion | 1. Orthodox Christian 2. Muslim 3. Catholic 4. Protestant 5. Others specify ------------------------------- |
|  | Gravidity | 1. Primigravida  2. Multigravida |

**Risk factors associated with HIV/HCV co-infection among HBsAg positive delivering mothers attended governmental hospitals in Addis Ababa**

| s.no | Variables | Response |
| --- | --- | --- |
|  | History of Previous blood transfusion | 1. Yes 2. No |
|  | History of STD | 1. Yes 2.No |
|  | History of previous abortion | 1. Yes 2. No |
|  | History of surgical procedure | 1. Yes 2. No |
|  | History of dental procedure | 1. Yes 2. No |
|  | History of tattooing | 1. Yes 2.No |
|  | History of ear piercing | 1. Yes 2. No |
|  | History of nose piercing | 1. Yes 2. No |
|  | History of home delivery by traditional birth attendants | - 1. Yes 2. No |
|  | History of having multiple sexual partners | 1. Yes 2. No |
|  | History of female circumcision | 1. Yes 2. No |
|  | History of hospital admission | 1. Yes 2. No |
|  | Presence of hepatitis B infected person in a family | 1. Yes 2. No |
|  | History of sharing shavers, razors, or earrings (at homes, beauty salon or, barber shops) | - 1. Yes   2. No |
|  | History of sharing tooth brushes with others | - 1. Yes 2. No |
|  | History of jaundice | 1. Yes 2. No |
|  | Contact with jaundice patient | 1. Yes 2. No |

**HIV and HCV status of the delivering mother**

1. HIV status 1. Positive 2. Negative

2. anti-HCV status 1. Positive 2. Negative

የተሳታፊው አጠቃላይ ሁኔታ

የጤና ተቓሙ ስም---------------------------

| ተ.ቁ | መለክያ | ምላሽ |
| --- | --- | --- |
| 1. | ዕድሜ(በዓመት) |  |
| 2. | የጋብቻ ሁኔታ | - - - 1. ያገባች       2. ያላገባች       3. የተፋታች       4. የሞተባት       5. ሌላ ካለ ይገለፅ____________________ |
| 3. | የትምህርት ደረጃ | 1.ምንም ያልተማረች   1. አንደኛ ደረጃ ያጠናቀቀች (1-8) 2. ሁለተኛደረጃ ያጠናቀቀች (9-12) 3. ኮሌጅ ና ከዛ በላይ |
| 4 | የሥራ ሁኔታ | 1. የ መንግስት  2. የ ግል ድርጅት  3. መንግሰታዊ ያል ሁነ ድርጅት  4. የግል ስራ  5. ስራ አጥ  6. የ ቤት እመቤት  7. የ ቀን ሰራተኛ  8. ሌላ ካለ ይገለፅ________________________ |
| 5 | ሀይማኖት | - - - 1. ኦርቶዶክስ  1. ሙስሊም 2. ካቶሊክ 3. ፕሮቴስታንት 4. ሌላ ካለ ይገለፅ ---------- |
| 1.8 | እርግዝናሽ ለ ስንተኛ ግዜ ነው | 1.የመጀመርያ  2. ሁለተኛ ከዛ በላይ |

ሄፕታይተስ ቢ ተጠቂ ለሆኑ ወላጅ እናቶች ለተጨማሪ የኤች አይቪ ወይም ሄፓታይተስ ሲ በሽታ መጋለጥ ምክንያት የሆኑ ነገሮች

| ተ.ቁ | መለክያዎች | ምላሽ |
| --- | --- | --- |
| 1. | የ ሌላ ሰው ደም ተሰጥቶት ያውቃል? | 1.አዎ 2. አያውቅም |
| 2. | የ አባለዘር በሽታ ተጠቂ ሆነው ያውቃሉ ? | 1.አዎ 2.አላውቅም |
| 3. | ፅንስ የማቃረጥ ሁኔታ አጋጥሞት ያውቃል ? | 1.አዎ 2.አያውቅም |
| 4. | የቀዶ ጥገና ህክምና አድርገው ያውቃሉ ? | 1.አዎ 2.አላውቅም |
| 5. | የ ጥርስ ነቅለ ተከላ ህክምና አድርገው ያውቃሉ? | 1.አዎ 2.አላውቅም |
| 6. | ንቅሳት አከናውነው ያውቃሉ? | 1.አዎ 2. አላውቅም |
| 7. | ጆሮዎን ተበስተው ያውቃሉ ? | 1.አዎ 2. አላውቅም |
| 8. | አፍንጫዎን ተበስተው ያውቃሉ? | 1.አዎ 2. አላውቅም |
| 9. | በቤት ውስጥ በልምድ አዋላጅ ታግዘው ወልደው ያውቃሉ? | 1. አዎ 2. አላውቅም |
| 10. | ከ አንድ በላይ የወሲብ ጋደኛ ኑሮት ያውቓል? | 1.አዎ 2. አያውቅም |
| 11. | ተገርዘዋል ? | 1.አዎ 2. አልተገረዝኩም |
| 12. | በህመም ምክንያት ሆስፒታል ተኝተው ያውቃሉ ? | 1.አዎ 2. አላውቅም |
| 13. | በ ቤተሰባችሁ ውስጥ በሄፓታይተስ ቢ ቫ ይረስ የተያዘ ሰው አለ? | 1.አዎ 2. የለም |
| 14. | የ ጋራ መላጫ ዕቃዎች፣ምላጮች ወይም የጆሮ ጌጦችን ተጠቅመው ያውቃሉ(በ ቤት ውስጥ፣በ ውበት ሳሎን ) | 1.አዎ 2.አላውቅም |
| 15. | የ ጋራ ጥርስ ብሩሽ ተጠቅመው ያውቃሉ? | 1.አዎ 2. አላውቅም |
| 16. | ዓይንዎን ጥፍርዎን ወይም ቆዳዎትን ብጫ የመሆን ነገር አጋጥሞት ያውቃል | 1. አዎ 2. አላጋጠመኝም |
| 17. | ዓይኑን ጥፍሩን ወይም ቆዳውን ብጫ የመሆን ነገር ካጋጠመው ህመምተኛ ጋር ቅርበት ኑሮት ያውቃል ? | 1.አዎ 2. አያውቅም |

በደምዎ ውስጥ የኤች አይቪ ወይም ሄፓታይተስ ሲ ቫይረስ መኖር ወይም አለመኖር ሁኔታ

- 1. ኤች አይቪ 1. አለ 2.የለም
  2. ሄፓታይተስ ሲ 1.አለ 2. የለም
