# Supplementary material for: Co-infection of HIV or HCV among HBsAg positive delivering mothers and its associated factors in governmental hospitals in Addis Ababa, Ethiopia: A cross-sectional study
Source: PLoS One. 2022 Aug 26;17(8):e0273300. doi: 10.1371/journal.pone.0273300 (PMC9417033; doi:10.1371/journal.pone.0273300)
Supplement: Attachment — Submitted filename: PONE-D-21-13799_reviewer (1).pdf [file pone.0273300.s002.pdf]

PLOS ONE

Co-infection of HIV or HCV among HBsAg positive delivering mothers and its associated factors in governmental hospitals in Addis Ababa, Ethiopia: a cross-sectional study

--Manuscript Draft--

**Manuscript Number:**	PONE-D-21-13799
**Article Type:**	Research Article
**Full Title:**	Co-infection of HIV or HCV among HBsAg positive delivering mothers and its associated factors in governmental hospitals in Addis Ababa, Ethiopia: a cross-sectional study
**Short Title:**	co-infection of HIV or HCV among HBsAg positive delivering mothers
**Corresponding Author:**	Mebrihit Arefaine Tesfu, MSC<br>Addis Ababa University<br>Addis Ababa, Addis Ababa ETHIOPIA
**Keywords:**	HBV; HIV; HCV, HBV-HIV co-infection; HBV-HCV co-infection; pregnant women; delivering mothers

**Abstract:**	Background<br><br>Blood borne viral infections such as hepatitis B virus (HBV), Hepatitis C virus (HCV), and Human immunodeficiency virus (HIV) cause substantial mortality and morbidity worldwide. Viral hepatitis during pregnancy is closely related to high risks of maternal and neonatal complications. In Ethiopia, only a little information is available on co-infection of HCV or HIV among Hepatitis B surface Antigen (HBsAg) positive pregnant mothers. The study aimed to determine HIV or HCV co-infection and associated risk factors among HBsAg positive delivering mothers.<br><br>Method<br><br>A health facility-based cross-sectional study was conducted in five governmental hospitals in Addis Ababa among 265 HBsAg –positive delivering mothers in the year 2019and 2020. A purposive sampling technique was used to select the study participants. Structured questioners and laboratory test results were used to collect the data. SPSS version 20 software was used to enter and analyzed the data. Multivariable logistic regression was used to identify independent predictors of HIV or HCV co-infections.<br><br>Results<br><br>Of the HBsAg positive delivering mothers, 9(3.4%) and 3(1.1%) were co-infected with HIV and HCV, respectively. None of the socio-demographic characteristics were significantly associated with these viral co-infections. History of sexually transmitted diseases (AOR=9.3; 95% CI: 1.84-47.1), multiple sexual partners (AOR== 5.96; 95% CI: 1.074-33.104), and history of sharing shavers, razors, and earrings (AOR=5.5;95%CI: 1.014-29.69) were significantly associated with HIV co-infection. HCV-co-infection was not significantly associated with any of the potential risk factors.<br><br>Conclusion<br><br>This dual infection rate of HBsAg positive delivering mother with HIV or HCV indicates that a substantial number of infants born in Ethiopia are at high risk of mother-to-child transmission of these viruses. Thus, all pregnant mothers need to be screened for HBV, HCV, and HIV during antenatal care, and also it needs implementation of prevention mechanisms of mother-to-child transmissions of these viral infections.

**Order of Authors:**	Mebrihit Arefaine Tesfu, MSC
	Nega Berhe Belay
	Tilahun Teklehaymanot Habtemariam

Powered by Editorial Manager® and ProduXion Manager® from Aries Systems Corporation

Additional Information:	
**Question**	**Response**
**Financial Disclosure**<br><br>Enter a financial disclosure statement that describes the sources of funding for the work included in this submission. Review the submission guidelines for detailed requirements. View published research articles from *PLOS ONE* for specific examples.<br><br>This statement is required for submission and **will appear in the published article** if the submission is accepted. Please make sure it is accurate.<br><br>**Unfunded studies**<br>Enter: *The author(s) received no specific funding for this work.*<br><br>**Funded studies**<br>Enter a statement with the following details:<br>• Initials of the authors who received each award<br>• Grant numbers awarded to each author<br>• The full name of each funder<br>• URL of each funder website<br>• Did the sponsors or funders play any role in the study design, data collection and analysis, decision to publish, or preparation of the manuscript?<br>• **NO** - Include this sentence at the end of your statement: *The funders had no role in study design, data collection and analysis, decision to publish, or preparation of the manuscript.*<br>• **YES** - Specify the role(s) played.<br><br>* typeset	The author(s) received no specific funding for this work.
**Competing Interests**<br><br>Use the instructions below to enter a competing interest statement for this submission. On behalf of all authors, disclose any competing interests that could be perceived to bias this work—acknowledging all financial support and any other relevant financial or non-financial competing interests.	The authors have declared that no competing interests exist.

Powered by Editorial Manager® and ProduXion Manager® from Aries Systems Corporation

This statement is required for submission and **will appear in the published article** if the submission is accepted. Please make sure it is accurate and that any funding sources listed in your Funding Information later in the submission form are also declared in your Financial Disclosure statement.<br><br>View published research articles from *PLOS ONE* for specific examples.<br><br>**NO authors have competing interests**<br><br>Enter: *The authors have declared that no competing interests exist.*<br><br>**Authors with competing interests**<br><br>Enter competing interest details beginning with this statement:<br><br>*I have read the journal's policy and the authors of this manuscript have the following competing interests: [insert competing interests here]*<br><br>* typeset	
**Ethics Statement**<br><br>Enter an ethics statement for this submission. This statement is required if the study involved:<br><br>• Human participants<br>• Human specimens or tissue<br>• Vertebrate animals or cephalopods<br>• Vertebrate embryos or tissues<br>• Field research<br><br>Write "N/A" if the submission does not require an ethics statement.<br><br>General guidance is provided below. Consult the submission guidelines for detailed instructions. **Make sure that all information entered here is included in the**	Ethical clearance and approval were obtained from the Institutional Review Board of Aklilu Lemma Institute of Pathobiology, Addis Ababa University, and Addis Ababa Health Bureau. Permission to carry out the study was obtained from the health facilities .After explaining the purpose, written informed consent was obtained from the delivering mothers. Moreover, confidentiality was assured for all the information provided and, personal identifiers were not included in questionnaires.

**Methods section of the manuscript.**<br><br>**Format for specific study types**<br><br>**Human Subject Research (involving human participants and/or tissue)**<br>• Give the name of the institutional review board or ethics committee that approved the study<br>• Include the approval number and/or a statement indicating approval of this research<br>• Indicate the form of consent obtained (written/oral) or the reason that consent was not obtained (e.g. the data were analyzed anonymously)<br><br>**Animal Research (involving vertebrate animals, embryos or tissues)**<br>• Provide the name of the Institutional Animal Care and Use Committee (IACUC) or other relevant ethics board that reviewed the study protocol, and indicate whether they approved this research or granted a formal waiver of ethical approval<br>• Include an approval number if one was obtained<br>• If the study involved *non-human primates*, add *additional details* about animal welfare and steps taken to ameliorate suffering<br>• If anesthesia, euthanasia, or any kind of animal sacrifice is part of the study, include briefly which substances and/or methods were applied<br><br>**Field Research**<br><br>Include the following details if this study involves the collection of plant, animal, or other materials from a natural setting:<br>• Field permit number<br>• Name of the institution or relevant body that granted permission	
**Data Availability**<br><br>Authors are required to make all data underlying the findings described fully available, without restriction, and from the time of publication. PLOS allows rare exceptions to address legal and ethical concerns. See the PLOS Data Policy and FAQ for detailed information.	No - some restrictions will apply

A Data Availability Statement describing where the data can be found is required at submission. Your answers to this question constitute the Data Availability Statement and **will be published in the article**, if accepted.<br><br>**Important:** Stating 'data available on request from the author' is not sufficient. If your data are only available upon request, select 'No' for the first question and explain your exceptional situation in the text box.<br><br>Do the authors confirm that all data underlying the findings described in their manuscript are fully available without restriction?	
**Describe where the data may be found in full sentences. If you are copying our sample text, replace any instances of XXX with the appropriate details.**<br><br>• If the data are **held or will be held in a public repository**, include URLs, accession numbers or DOIs. If this information will only be available after acceptance, indicate this by ticking the box below. For example: *All XXX files are available from the XXX database (accession number(s) XXX, XXX.*).<br>• If the data are all contained **within the manuscript and/or Supporting Information files**, enter the following: *All relevant data are within the manuscript and its Supporting Information files.*<br>• If neither of these applies but you are able to provide **details of access elsewhere**, with or without limitations, please do so. For example:<br><br>*Data cannot be shared publicly because of [XXX]. Data are available from the XXX Institutional Data Access / Ethics Committee (contact via XXX) for researchers who meet the criteria for access to confidential data.*<br><br>*The data underlying the results*	All relevant data are within the manuscript and its Supporting Information files.

presented in the study are available from (include the name of the third party and contact information or URL).<br>• This text is appropriate if the data are owned by a third party and authors do not have permission to share the data.<br><br>* typeset	
Additional data availability information:	

Powered by Editorial Manager® and ProduXion Manager® from Aries Systems Corporation

Manuscript

Click here to access/download;Manuscript;manuscript on HBVHIVHCV.docx

Co-infection of HIV or HCV among HBsAg positive delivering mothers and its associated factors in governmental hospitals in Addis Ababa, Ethiopia: a cross-sectional study

Mebrihit Arefaine Tesfu [1]*, Nega Berhe Belay [1], Tilahun Teklehaymanot Habtemariam[1]

[1]Aklilu Lemma Institute of pathobiology , Addis Ababa Universty , Addis Ababa,Ethiopia

* Corresponding author: mebrihitarefaine@gmail.com(MAT)

Abstract

Background: Blood borne viral infections such as hepatitis B virus (HBV), Hepatitis C virus (HCV), and Human immunodeficiency virus (HIV) cause substantial mortality and morbidity worldwide. Viral hepatitis during pregnancy is closely related to high risks of maternal and neonatal complications. In Ethiopia, only a little information is available on co-infection of HCV or HIV among Hepatitis B surface Antigen (HBsAg) positive pregnant mothers. The study aimed to determine HIV or HCV co-infection and associated risk factors among HBsAg positive delivering mothers.

Method: A health facility-based cross-sectional study was conducted in five governmental hospitals in Addis Ababa among 265 HBsAg –positive delivering mothers in the year 2019 and 2020. A purposive sampling technique was used to select the study participants. Structured questioners and laboratory test results were used to collect the data. SPSS version 20 software was used to enter and analyzed the data. Multivariable logistic regression was used to identify independent predictors of HIV or HCV co-infections.

Results: Of the HBsAg positive delivering mothers, 9 (3.4 %) and 3 (1.1%) were co-infected with HIV and HCV, respectively. None of the socio-demographic characteristics were significantly associated with these viral co-infections. History of sexually transmitted

diseases (AOR=9.3; 95 % CI: 1.84-47.1), multiple sexual partners (AOR== 5.96; 95% CI: 1.074-33.104), and history of sharing shavers, razors, and earrings (AOR=5.5;95%CI: 1.014-29.69) were significantly associated with HIV co-infection. HCV-co-infection was not significantly associated with any of the potential risk factors.

Conclusion: This dual infection rate of HBsAg positive delivering mother with HIV or HCV indicates that a substantial number of infants born in Ethiopia are at high risk of mother-to-child transmission of these viruses. Thus, all pregnant mothers need to be screened for HBV, HCV, and HIV during antenatal care, and also it needs implementation of prevention mechanisms of mother-to-child transmissions of these viral infections.

Background

Blood-borne viral infections such as hepatitis B virus, Hepatitis C virus, and Human immunodeficiency virus cause substantial mortality and morbidity worldwide [1].

Need to join th
paragraphs tog

Hepatitis B virus (HBV) infection occurs globally and constitutes a major public health problem. It infects over 20 million people globally every year, and there are around 350-400 million chronic carriers. More than 1.2 million deaths occur annually from HBV-related disease, making it the 10th leading cause of death, and the second most common cause of cancer deaths after tobacco, globally[2,3]. HBV is an infectious disease, and it mainly transmits through mother-to-child, skin and mucous membrane infections by contaminated blood or body fluids, sexual contacts, and injection drug abuse. Besides, body tattooing, ear piercing, acupuncture, dialysis, and even using a syringe can be the source of infection [4]. HBV acquisition in adulthood commonly leads to acute resolved infection and immunity. But, perinatal-/neonatal HBV infection more likely leads to chronic infection and its long-term disease risks [5].

Worldwide the prevalence of ~~Hepatitis C Virus~~ infection in pregnant women and children has been estimated ~~to~~ 1-8% and 0.05-5%, respectively. ~~Even though~~, ~~D~~irect percutaneous inoculation is the most efficient mode of transmission of HCV, sexual, household, occupational, and vertical transmission may also be important[6,7,8].Mother-to-child transmission of HCV increases to 4–25% times if the mother is also ~~HIV infected~~[9].

Over the last decades, ~~the Human immunodeficiency virus (HIV)~~ infection has been one of the largest public health challenges, especially in low and middle-income countries [7]. Among the HIV-~~infected~~ patients, 2-4 million are estimated to have chronic HBV co-infection, and 4-5 million are co-infected with HCV [10].HIV in pregnancy has adverse outcomes to maternal and fetal health and also to health workers at times of delivery [7] .

HBV, HIV, and HCV have similarities in mode of transmission ~~(11, 12). But~~ there are slight variations in the mode of transmission of these viruses. Unlike HIV, HBV is not transmitted by breastfeeding, furthermore, child to child transmission is common for HBV but not for HIV. HBV is 50–100 times and 10 times more infectious than HIV and HCV, respectively [7].The presence of higher concentrations of HBV in body fluids of persons with acute or chronic HBV infection than persons with HIV infection may cause HBV more infectious than HIV[11].

Co-infection of ~~both hepatitis viruses~~ with HIV is associated with low CD4 count, accelerated liver disease progression, higher mortality, and mother-to-child transmission of the viruses [7,13.14 and15]. Additionally, the progression rate and complications such as liver fibrosis, cirrhosis, end-stage liver disease, hepatocellular carcinoma (HCC) and mortality due to liver pathology arising from HBV infection are accelerated in patients with HIV co-infected patients than in patients with HBV infection alone[7,16–].The clinical management of individuals co-infected with those viruses is challenging[17].

Available data suggest that, in resource-rich settings, approximately 10% of the HIV-infected population have chronic HBV infection and around a third have chronic HCV infection [13, 16]. However, wide regional variations are observed with co-infection of HIV and HBV prevalence rates estimated to be 5–10 % in areas such as North America, Europe and Australia compared to higher prevalence rates of 20–30 % in areas such as Sub-Saharan Africa and Asia [16].

HBV/HIV co-infection rates of 12.9 % [18] and 16% [19] were reported in people living with HIV in Cameroon. Besides, HBV/HIV Co-infection rates of 11.8% [20] and 3.1% [21] were reported in pregnant mothers in Nigeria and South Africa, respectively. HCV/HIV co-infection rates of 0%-33% were reported in pregnant mothers in Nigeria [22-24].

HBV/HIV co-infection rates of 19%-40% were reported among pregnant mothers according to different studies conducted in Ethiopia [7, 25 and 26]. Hundred percent of HCV positive pregnant mothers were also co-infected with HIV in Atat Hopsital, Southern, Ethiopia[7] and 2.9% of HCV/HIV co-infection was reported in pregnant mothers in East Wollega Zone, West Oromia, Ethiopia[9] .None of the pregnant mothers were co-infected with HBV and HCV[27,28] .

Several studies were conducted to determine co-infection of HBV-HIV, HBV- HCV or HIV-HCV among the general population, the general pregnant mothers, or HIV-infected pregnant mothers[7,13,16,18-28], but only a few studies have been conducted to determine co-infection of HIV or HCV and related risk factors among HBsAg positive delivering mothers globally and particularly in Ethiopia. The study aimed to assess the co-infection of these viruses among HBsAg positive delivering mothers and factors associated.

Methods and materials

delete the extra spacing between these headings and paragraphs

Study design and period: a health facility based cross -sectional study design was employed from January 2019 up to December 2020.

Study site and population: The study was conducted in Addis Ababa, the capital city of Ethiopia; with an estimated population of 7.8236 million in 2019 (29).The city has a higher population of female occupants as compared to male inhabitants. In the city, there are 14 governmental hospitals and 103 health centers. In addition to this, there are many private hospitals and clinics which provide health service for the community of the city and patients from other parts of the country. The study was conducted in 5 of the governmental hospitals that have maternal and child health care services. The hospitals were Zewditu Memorial Hospital, Gandhi Memorial Hospital, Yekatit 12 Hospital Medical College, Armed force Hospital and Menelik II referral Hospital. The study population was HBsAg positive delivering mothers who attended in the selected governmental hospitals in Addis Ababa.

Sample size determination and sampling technique

A single population proportion formula was used. The sample size (n) of this study was determined using 22.2 % of HBV/HIV co-infection in pregnant women in Addis Ababa [26] and giving any particular outcome to be with 5 % marginal error and 95% confidence interval. Based on this assumption, the actual sample size for this study was computed using one sample population proportion formula indicated below

$$n = (z\,\partial/2)^{2}\, p\,(1-p)\,/d^{2}$$

Where n = sample size P= 0.222, d = 0.05(5% error of margin) z ∂/2 = 1.96 (standard normal probability for 95% CI) n = 265

After identifying governmental health facilities that have maternal and child health services, study health facilities were selected using the simple random sampling method. A purposive sampling technique was employed to select the study participants.

Eligibility

Inclusion criteria: All HBsAg positive delivering mothers who attended the selected hospitals in Addis Ababa and those who were volunteer to participate and give informed consent were included.

Exclusion criteria: Delivering mothers who had communication problems were excluded.

Study variables

Dependent variables: co-infection of HIV among HBsAg positive delivering mothers, Co-infection of HCV among HBsAg positive delivering mothers.

Independent variables: age, marital status, educational status, occupation, religion, gravidity, history of blood transfusion, history of Sexually transmitted diseases(STD), history of abortion, history of surgical procedure, history of dental procedure, history of tattooing, history of ear piercing, history of nose piercing, history of home delivery by traditional birth attendants, history of multiple sexual partners, history of female circumcision, history of hospital admission, Presence of known hepatitis B infected person in a family, history of sharing shavers, razors, or earrings (at homes, beauty salon or, barber shops), history of sharing toothbrushes with others, history of direct contact with contaminated medical devices, history of jaundice and history of contact with jaundice patients.

Data collection

A questionnaire ~~that~~ was originally prepared in English translated into Amharic and back to English that was interviewed by the data collectors and blood samples were used.

Data collection procedures and quality assurance

Delivering mothers who were diagnosed ~~positive for~~ HBsAg during screening for the virus as routine antenatal care service were selected based on the status on their medical records. To get information on socio-demographic characteristics of the respondents and risk factors associated with co-infection of the viruses, pre-designed and pretested structured questioners were used. Ten BSC ~~M~~idwives and Medical laboratory technologists working in the selected hospital were collected the data. Five supervisors with a second degree in health-related fields who recruited based on their experience in data collection and supervision participated. Two days of training were given on the objective of the study, obtaining consent, confidentiality of the information and~~,~~ data collection procedure for the data collectors and supervisors. Questionnaires were carefully designed and pre-tested with individuals' equivalent to 5% of the calculated sample size in Ras Desta Damtew hospital. Questionnaires were slightly adjusted after pretested results show a lack of clarity. To determine the co-infection of the viruses, 5 ml of venous blood was collected based on the standard collection procedure and placed in ethylene-diamine-tetra-acetic acid (EDTA) tubes. These tubes were labelled with unique identification number and processed at the time of collection. The supervisors and the principal investigator ~~have~~ supervised the data collection process.

Laboratory procedures

The blood samples taken from the individuals were centrifuged at 3000 revolutions per minute (RPM) for at least 10 minutes at room temperature. Then the plasma was tested for HBsAg (to ~~re-check~~ the status) and anti-HCV using separated rapid test Cassettes (Nantong

diagnosis biotechnology co.Ltd P.R.china), which have specificity and sensitivity of greater than 99%.Testing was done following the manufacturer's protocol. The HIV status of the delivering mothers was used from the antenatal care records since the test is performed on a routine basis.

Data management and analysis

The generated data was cleaned, coded, and uploaded into a computer using SPSS version 20.0 statistical software for analysis and interpretation. Descriptive values were expressed as the frequency, percentage, and mean ± standard deviation (SD). Logistic regression analysis was implemented to explore and determine the relationship of predictors on outcome variables. Variables significant at $p < 0.25$ with the dependent variable were selected for multivariable analysis. Odds ratio with 95% confidence level was computed, and a significant association was declared at $p < 0.05$.

Ethical consideration

Ethical clearance and approval were obtained from the Institutional Review Board of Aklilu Lemma Institute of Pathobiology, Addis Ababa University, and Addis Ababa Health Bureau. Permission to carry out the study was obtained from the health facilities .After explaining the purpose, written informed consent was obtained from the delivering mothers. Moreover, confidentiality was assured for all the information provided and, personal identifiers were not included in questionnaires. Results were reported to physicians for treatment management and prevention of mother-to-child transmission of these viral infections.

Results

Socio demographic characteristics of the study participants

A total of 265 HBsAg positive delivering mothers who attended governmental hospitals in Addis Ababa were included. The mean age of the study participants was 28.04 years with a standard deviation of 4.62 (SD). The majority of the delivering mothers (43.1 %) were in the age group of 25-29 years. Most (92.5%) of the delivering mothers were married. About 45.3 % of the mothers were housewives followed by 19.6% and 17.7% private employees and, government employees, respectively. Respondents were predominantly Orthodox Christians (71.7%) followed by Muslims (16.6%) and Protestants (9.8%).More than half (60 %) were multigravida mothers (Table1).

Table 1: socio-demographic characteristics of HBsAg positive delivering mothers in governmental hospitals in Addis Ababa, 2019-2020

**Variable**	**Category**	Number	**Percentage**
Age	<20	4	1.5
	20-24	61	23
	25-29	115	43.4
	30-34	60	22.6
	≥35	25	9.4
Marital status	Married	245	92.5
	Single	12	4.5
	Divorced	8	3
Educational status	No formal education	28	10.6
	Primary level(1-8)	98	37
	Secondary level(9-12)	75	28.3
	College diploma and above	64	24.2
Occupation	Government employee	47	17.7
	Private employee	52	19.6
	Self- employee	27	10.2
	House wife	120	45.3
	Others	19	7.2
Religion	Orthodox	190	71.7
	Muslim	44	16.6
	Protestant	26	9.8
	Others	5	1.9
Gravidity	Primigravida	106	40
	Multigravida	159	60

Prevalence of HIV co-infection among HBsAg positive delivering mothers

Out of the 265 HBsAg positive delivering mothers, 9(3.4%) were co-infected with HIV. Of those77.8% were married and multigravida mothers and, 66.7% were ~~in the age group of~~ 25-29 years old and orthodox Christians. There were no significant differences in HIV co-infection prevalence among all socio-demographic characteristics of the study participants (Table 2).

Table2: Socio-demographic characteristics and HIV co- infection among HBsAg positive delivering mothers in governmental hospitals in Addis Ababa, 2019-2020.

**Variable**	**Category**	HIV status		COR(95%CI)	P value
		Positive(9) n(%)	Negative(256) n(%)		
Age	<20	0	4(1.6)	1.39(9.658-2.951)	0.386
	20-24	2(22.2)	59(23)		
	25-29	6(66.7)	109(42.6)		
	30-34	1(11.1)	59(23)		
	≥35	0	25(9.8)		
Marital status	Married	7(77.8)	238(93)	4.86(.524-44.9)	0.164
	Single	1(11.1)	11(4.3)	1.57(.084-29.4)	0.762
	Divorced	1(11.1)	7(2.7)	1	
Educational status	Illiterate	2(22.2)	26(10.2)	.206(0.18-2.38)	0.206
	Primary level	4(44.4)	94(36.7)	.373(.041-3.42)	0.383
	Secondary level	2(22.2)	73(28.5)	.579(.051-6.54)	0.659
	College diploma and above	1(11.1)	63(24.6)	1	
Occupation	Government employee	1(11.1)	46(18)	5.75(.488-67.8)	0.165
	Private employee	2(22.2)	50(19.5)	3.13(.407-24.01)	0.273

	Self- employee	1(11.1)	27(10.5)	3.34(.283-40.25)	0.336
	House wife	3(33.3)	117(45.7)	4.88(.756-31.44)	0.096
	Others	2(22.2)	16(6.3)	1	
Religion	Orthodox	6(66.7)	184(71.9)	7.68(.741-79.34)	0.088
	Muslim	1(11.1)	43(16.8)	10.75(.560-206.4)	0.115
	Protestant	1(11.1)	25(9.8)	6.25(.322-121.33)	0.226
	Others	1(11.1)	4(1.6)	1	
Gravidity	Primigravida	2(22.2)	104(40.6)	2.39(.488-11.757)	0.282
	Multigravida	7(77.8)	152(59.4)	1	

Risk factors associated with HIV co-infection among HBsAg positive delivering mothers

Most of (88.9%) the HIV co-infected mothers had a history of ear piercing and, 44.4% of them had a history of sexually transmitted infection, previous abortion, and female circumcision. About 7(77.8%) and 6(66.7%) of them had history of having multiple sexual partners and sharing shavers, razors or earrings, respectively. In multivariable analysis, history of sexually transmitted diseases (STD), history of have having multiple sexual partners and history of sharing shaver, razor or earrings were considered as potential risk factors for HIV co-infection. HBsAg positive delivering mothers who had a history of sexually transmitted infections were 9.3 times more likely to have HIV co-infection compared to their counterparts (AOR=9.3; 95 % CI: 1.84-47.1). Mothers who had multiple sexual partners were 5.96 times more likely to have HIV co-infection (AOR= 5.96; 95% CI: 1.074-33.104). The odds of having HBV-HIV co-infection were 5.5 times higher among mothers who had a history of sharing shaver, razor and earrings (at homes, beauty salon or barbershops)(AOR=5.5;95%CI : 1.014-29.69)(Table3).

Table3: factors associated with HIV co-infection among HBsAg positive delivering mothers in governmental hospitals in Addis Ababa, 2019-2020

Variables	Category	HIV status		Bivariate analysis		Multivariate analysis	
				P value	COR(95%CI)	AOR(95%CI)	P value
		Positive(9) n(%)	Negative(256) n(%)				
History of blood transfusion	Yes	1(11.1)	16(6.3)	0.565	1.88(.221-15.93)	NA	
	No	8(88.9)	239(93.7)		1		
History of STD	Yes	4(44.4)	11(4.3)	0.000	17.82(4.19-75.7)	9.3(1.84-47.1)	0.007
	No	5(55.6)	246(95.7)		1	1	
History of abortion	Yes	4(44.4)	44(17.2)	0.051	3.86(.995-14.9)	2.69(.539-13.44)	0.227
	No	5(55.6)	212(82.8)		1	1	
History of surgical procedure	Yes	1(11.1)	27(10.6)	0.926	1.11(.133-9.194)	NA	
	No	8(88.9)	230(89.4)		1		
History of dental procedure	Yes	1(11.1)	37(14.5)	0.779	.74(.090-6.1)	NA	
	No	8(88.9)	219(85.5)		1		
History of tattooing	Yes	2(22.2)	44(17.2)	0.696	1.37(.277-6.58)	NA	
	No	7(77.8)	212(82.8)		1		
History of ear piercing	Yes	8(88.9)	249(97.3)	0.186	.23(.025-2.05)	0.26(.019-3.51)	0.308

	No	1(11.1)	7(2.7)		1	1	
History of nose piercing	Yes	1(11.1)	12(4.7)	0.397	2.54(.294-21.99)	NA	
	No	8(88.9)	244(95.3)		1		
History of home delivery by traditional birth attendants	Yes	1(11.1)	8(3.1)	0.226	3.88(.432-34.79)	0.83(.042-16.7)	0.905
	No	8(88.9)	248(96.9)		1		
History of having multiple sexual partners	Yes	7(77.8)	67(26.2)	0.005	5.53(1.345-22.73)	5.96(1.074-33.104)	0.041
	No	2(22.2)	189(73.8)		1		
History of female circumcision	Yes	4(44.4)	123(48)	0.692	1.31(.349-5.00)	NA	
	No	5(55.6)	133(52)		1		
History of hospital admission	Yes	2(22.2)	38(14.8)	0.552	1.69(.338-8.46)	NA	
	No	7(77.8)	218(85.2)		1		
Presence of known hepatitis B infected person in a family	Yes	2(22.2)	26(10.2)	0.263	2.53(.499-12.81)	NA	
	No	7(77.8)	230(89.8)		1		
History of sharing shavers, razors, or earrings (at homes, beauty salon or, barber shops)	Yes	6(66.7)	72(28.1)	0.022	5.21(1.269-21.40)	5.5(1.014-29.69)	0.048
	No	3(33.3)	184(71.9)		1	1	
History of sharing tooth brushes with others	Yes	1(11.1)	10(3.9)	0.311	3.08(.350-27.00)	NA	
	No	8(88.9)	246(96.1)		1		
History of jaundice	Yes	1(11.1)	12(4.7)	0.397	2.54(.294-	NA	

					21.988)		
	No	8(88.9)	244(95.3)		1		
History of contact with jaundice patient	Yes	1(11.1)	16(6.3)	0.565	1.88(.221-15.93)	NA	
	No	8(88.9)	240(93.7)		1		

CI: confidence interval

COR: Crude odds ratio, AOR: adjusted odds ratio

NA: not applicable refers to factors with p-value ≥0.25 at the bivariate analysis which were not considered in Multivariable analysis.

Prevalence of HCV co-infection among HBsAg positive delivering mothers

Only3 (1.1%) of the HBsAg Positive delivering mothers were co-infected with HCV. All of the HCV co-infected mothers were married. Two (66.7%) were ~~in the age group of~~ 25-29, primary educational level, orthodox Christian ~~followers~~ and multigravida. However, none of the socio-demographic characteristics were significantly associated with co-infection of HCV (Table 4).

Table4: **Socio-demographic characteristics and HCV co-infection among HBsAg positive delivering mothers in governmental hospitals in Addis Ababa, 2019-2020**

**Variable**	**Category**	HCV status		P value
		Positive(3) n(%)	Negative(262) n(%)	
Age	<20	0	4(1.5)	0.348
	20-24	0	61(23.8)	
	25-29	2(66.7)	113(44.1)	
	30-34	0	60(23.4)	
	≥35	1(33.3)	24(9.2)	
Marital status	Married	3(100)	245(93.5)	0.999
	Single	0	12(4.5)	
	Divorced	0	8(3.1)	
Educational status	Illiterate	0	28(10.9)	0.997
	Primary level	2(66.7)	96(37.5)	
	Secondary level	0	75(29.3)	
	College diploma and above	1(33.3)	63(20)	
Occupation	Government employee	0	47(18.4)	0.888
	Private employee	1(33.3)	51(19.5)	
	Self- employee	1(33.3)	26(9.9)	
	House wife	1(33.3)	119(46.5)	

	Others	0	19(7.3)	
Religion	Orthodox	2(66.7)	188(73.4)	0.128
	Muslim	0	44(17.2)	
	Protestant	0	26(9.9)	
	Others	1(33.3)	4(1.5)	
Gravidity	Primigravida	1(33.3)	105(40.1)	0.813
	Multigravida	2(66.7)	157(59.9)	

Risk factors associated with HCV co-infection among HBsAg positive delivering mothers

All (100%) of the HCV co-infected delivering mothers had a history of ear piercing, and 2(66.7%) had a history of tattooing, ~~history of~~ female circumcision, ~~history of~~ sharing shavers, razors, and earrings (at homes, beauty salon or, barber shops) and ~~history of~~ jaundice. About 1(33.3%) had a history of~~;~~ blood transfusion, previous abortion, surgical procedure, dental procedure, multiple sexual partners, and contact with jaundice patients. Even though the history of blood transfusion, ~~history of~~ tattooing, ~~history of~~ home delivery by traditional birth attendants, ~~history of~~ jaundice, and ~~history of~~ contact with jaundice patients were candidate variables for multivariate analysis (P<0.25), none of them were significantly associated with HCV co-infection in multivariate analysis(Table5)

Table5: statistical association of predictor variables with HCV co-infection among HBsAg positive delivering mothers in governmental hospitals in Addis Ababa, 2019-2020

Variables	Category	HCV status		Bivariate analysis		Multivariate analysis	
				P value	COR(95%CI)	AOR(95%CI)	P value
		Positive (3) n(%)	Negative(262) n(%)				
History of blood transfusion	Yes	1(33.3)	16(6.1)	0.103	7.68(.661-89.36)	1.98(.045-87.660	0.722
	No	2(66.7)	246(93.9)		1		
History of STD	Yes	0	15(5.7)				
	No	3(100)	247(94.3)				
History of abortion	Yes	1(33.3)	48(18.3)	0.526	2.23(.198-15.08)	NA	
	No	2(66.7)	214(81.7)		1		
History of surgical procedure	Yes	1(33.3)	27(10.3)	0.236	4.35(.382-49.6)		
	No	2(66.7)	235(89.7)				
History of dental procedure	Yes	1(33.3)	37(14.1)	0.369	3.04(.269-34.38)	NA	
	No	2(66.7)	225(85.9)		1		
History of tattooing	Yes	2(66.7)	42(16)	0.057	10.48(.929-118.17)	4.23(.222-80.790	0.338
	No	1(33.3)	220(84)		1		

History of ear piercing	Yes	3(100)	254(96.9)				
	No	0	8(3.1)				
History of nose piercing	Yes	0	13(5)				
	No	3(100)	249(95)				
History of home delivery by traditional birth attendants	Yes	1(33.3)	8(3.0)	0.030	15.88(1.30-193.68)	10.92(.179-665.05)	0.254
	No	2(66.7)	254(97)		1		
History of having multiple sexual partners	Yes	1(33.3)	73(27.9)	0.834	1.3(.116-14.5)	NA	
	No	2(66.7)	189(72.1)		1		
History of female circumcision	Yes	2(66.7)	99(37.8)	0.333	3.293(.295-36.79)	NA	
	No	1(33.3)	163(62.2)		1		
History of hospital admission	Yes	1(33.3)	38(14.5)	0.382	2.95(.261-33.3)	NA	
	No	2(66.7)	223(85.5)		1		
Presence of known hepatitis B infected person in a family	Yes	0	28(10.7)				
	No	3(100)	234(89.3)				
History of sharing shaver ,razor or earrings (at homes, beauty salon or, barber shops)	Yes	2(66.7)	75(28.6)	0.192	4.99(.445-55.32)	6.23(.292-133.3)	0.242
	No	1(33.3)	187(71.4)		1		
History of sharing tooth brushes with others	Yes	0	11(4.2)				
	No	3(100)	251(95.8)				

History of jaundice	Yes	2(66.7)	11(4.2)	0.002	45.64(3.84-542.4)	25.16(.979-646.82)	0.052
	No	1(33.3)	251(95.8)		1		
History of contact with jaundice patient	Yes	1(33.3)	16(6.1)	0.103	7.69(.661-89.37)	3.22(.104-99.43)	0.504
	No	2(66.7)	246(93.9)				

CI: confidence interval

COR: Crude odds ratio, AOR: adjusted odds ratio

NA: not applicable refers to factors with p-value ≥0.25 at the bivariate analysis which were not considered in Multivariable analysis

HIV and HCV co-infection among the HBsAg positive delivering mothers

None of the HBsAg- positive delivering mothers were co-infected with both HIV and HCV (Table 6).

Table 6: HIV and HCV co-infection among HBsAg positive delivering mothers in governmental hospitals in Addis Ababa, 2019-2020

HIV status	HCV status		p-value
	Positive	Negative	0.999
Positive	0	9	
Negative	3	253	

Discussion

In the present study, 3.4% and 1.1% of the HBsAg positive delivering mothers were co-infected with HIV and HCV, respectively. There is a scarcity of data on co-infection of HIV or HCV among HBsAg positive delivering mothers, and factors associated to compare with our findings. This is due to most of the previous studies were conducted on general or HIV-infected pregnant mothers.

The 3.4 % HIV co-infection prevalence in our study is similar to studies in South Africa (3.1%) [21] and Rwanda (4.1%) [11]~~. But~~ it was smaller than HBV and HIV co-infection found among pregnant women in Europe (4.9%) [17], Nigeria (11.8%) [20], Bahir Dar city, Ethiopia (19%) [25], Atat Hospital, Southern Ethiopia (40%) [7] and Addis Ababa, Ethiopia (22.2%) [26]. In contrast it was higher than studies reported in Nigeria (0.24%) [23] and 2% [30], Southern Ethiopia (0.6%) [31] and 0% in eastern Ethiopia [32]. Furthermore, The 1.1% HCV co-infection in this study is similar to the study in Pakistan (1.3%) [33]. But it is lower than a study in west Iran (7.9%) [10] and higher than a study in Ghana(0.6%)[34]. None of the pregnant mothers were co-infected with HBV and HCV in studies in Ethiopia [27, 28], Nigeria [23], and Sana'a, Yemen [35]. These variations might be due to differences in sampling method, laboratory tests used and~~,~~ cultural and behavioural practices. Even though all of the socio-demographic characteristics were not significantly associated with both HIV and HCV co-infections, the majority (66.7%) of the co-infected mothers were in the age group of 25-29 years old. This is in line with the study conducted in Nigeria [30].Seven (77.8 %) of the HIV co-infected and 66.7% of the HCV co-infected mothers were multigravida. This is supported by studies from Rwanda [13] and Nigeria [6].

In our study, ~~all and~~ majority of the potential risk factors were not significantly associated with HCV and HIV co-infections, respectively. This might be due to~~;~~ the low number of HBV-HIV and HBV-HCV co-infected study participants in our study, HBV ~~is more~~ infectious than HIV and HCV [11], or early childhood transmission of HBV rather than shared routes of HIV, HCV, and HBV in adulthood [21].

History of having ~~sexually transmitted diseases~~ was significantly associated with HIV co-infection. This is in agreement with studies in Rwanda[11]and Brazil [36] .This might be due to ~~sexually transmitted diseases~~ ~~can be~~ able to increase the susceptibility to infection by both viruses through mucosal disruption, immune changes, and microenvironment effects on the genital tract. History of having multiple sexual partners was another significant risk factor for the HBV-HIV co-infection. This is in agreement with studies in Southern Ethiopia [7], Rwanda [11] and~~,~~ Brazil [36].This might be due to both HIV and HBV are sexually transmitted infections, and the transmission increases with the number of sexual partners. The history of sharing shavers, razors, and earrings (at homes, beauty salons, or barbershops) was also another significant risk factor to HIV co-infection in our study. This is similar to a study in Indonesia, identified that HIV transmission is possible due to lack of cleanliness of shaving equipment [37]. This might be due to HIV and other blood born infections can be transmitted via the use of sharing sharp material.

Conclusion and recommendation

Of the HBsAg positive delivering mothers 3.4% and 1.1% were dual infected with HIV and HCV, respectively. This indicates that a substantial number of infants born in Ethiopia are at high risk of mother to child transmission of HBV, HIV and HCV. All of the socio-demographic characteristics of the study participants and majority of the potential risk factors

were not significantly associated with co-infection of the viruses. History of having sexually transmitted diseases, having multiple sexual partners, and sharing of shavers, razors, and earrings were potential risk factors significantly associated with co-infection of HIV. All pregnant mothers need to be screened for HBV, HCV, and HIV during antenatal care and also it needs, implementation of prevention mechanisms of mother- to- child transmissions of those viral infections. Furthermore, awareness creation on the transmission of those blood born viral infections is needed.

Strengths and limitations of the study

We include, a large number of HBsAg positive delivering mothers than the previous studies and, it can help to more determine the rate and potential risk factors of the co-infections. Confirmatory tests like ELISA and PCR were not used because of a lack of laboratory setup.

Acknowledgement

We gratefully acknowledge the study participants, data collectors, and supervisors who participated in data collection.

References

1. Nyirenda, M. et al. Prevalence of infection with hepatitis B and C virus and coinfection with HIV in medical inpatients in Malawi. ELISAVIER, Journal of Infection .2008,57: 72–77.
2. Yelemkoure, E. T. et al.Prevention of mother-to-child transmission of hepatitis B virus in Burkina Faso: Screening, vaccination and evaluation of post-vaccination antibodies against hepatitis B surface antigen in newborns. Journal of Public Health in Africa .2018,9(3). doi: 10.4081/jphia.2018.816.
3. Rajendiran,S., Gopalan, U. and Jayakumar, K. Rajendiran Seroprevalence of Hepatitis B infection among pregnant women in South India. International Journal of Reproduction, Contraception, Obstetrics and Gynecology.2016, 6(1):249. doi: 10.18203/2320-1770.ijrcog20164668.
4. Mesfin, Y. M. and Kibret, K. T. Assessment of knowledge and practice towards hepatitis B among medical and health science students in Haramaya University, Ethiopia. PLoS ONE.2013,8(11): 1–6. doi: 10.1371/journal.pone.0079642.
5. Dionne-Odom, J., Tita, A. T. N. and Silverman, N. S.Hepatitis B in pregnancy screening, treatment, and prevention of vertical transmission.American Journal of Obstetrics and Gynecology92016). Elsevier Inc., 214(1) 6–14. doi: 10.1016/j.ajog.2015.09.100.
6. Gasim, G. I., Murad, I. A. and Adam, I. Regional Review Hepatitis B and C virus infections among pregnant women in Arab and African countries. J Infect Dev Ctries. 2013,7(8):566-578. doi: 10.3855/jidc.3243.
7. Bafa, T. A and Egata,A.D.Seroepidemiological patterns and predictors of hepatitis B , C and HIV viruses among pregnant women attending antenatal care clinic of Atat HospitaL,SouthernEthiopia.SAGEOpenMedicine.2020,8:19doi:10.1177/205031211990

0870.

8. Zhao, L., Daw, M. A. and Shah, K. B. Hepatitis C Virus: Molecular Pathways and Treatments. OMICS GroupeBooks.2014: 3–13.
9. Dabsu, R. and Ejeta, E.Seroepidemiology of Hepatitis B and C Virus Infections among Pregnant Women Attending Antenatal Clinic in Selected Health Facilities in East Wollega Zone, West Oromia, Ethiopia. BioMed Research International. 2018. doi: 10.1155/2018/4792584.
10. Mohammadi, M. et al .Survey of both hepatitis B virus (HBsAg) and hepatitis C virus (HCV-Ab) coinfection among HIV positive patients.BMCVirology journal.2009,6(202): 1–5. doi: 10.1186/1743-422X-6-202.
11. Mutagoma, M. et al. Hepatitis B virus and HIV co-infection among pregnant women in Rwanda. BMC Infectious Diseases. 2017, 17(1): 1–7. doi: 10.1186/s12879-017-2714-0.
12. Abatenh, E. et al.Hepatitis B Co - Infection in HIV AIDS Patients in Woldia, Ethiopia.Biomedical Journal of Scientific & Technical Research.2018, 10(3): 43–46. doi: 10.26717/bjstr.2018.10.001949.
13. Benhammou, V. et al. HBV or HCV Coinfection in HIV-1-Infected Pregnant Women in France: Prevalence and Pregnancy Outcomes', Journal of Acquired Immune Deficiency Syndromes.2018, 77(5): 439–450. doi: 10.1097/QAI.0000000000001618.
14. Mave, V. et al.Impact of maternal hepatitis B virus coinfection on mother-to-child transmission of HIV. HIV Medicine .2014, 15; 347–354 . doi: 10.1111/hiv.12120.
15. Matthews PC, Geretti AM, Goulder PJR , Klenerman P. Epidemiology and impact of HIV coinfection with Hepatitis B and Hepatitis C viruses in Sub-Saharan Africa. Journal of Clinical Virology.2014, 61: 20–33.
16. Agyeman AA and Ofori‑Asenso R.Prevalence of HIV and hepatitis B coinfection in Ghana: a systematic review and meta‑analysis. AIDS Res Ther.2016, 13:23 DOI

10.1186/s12981‑016‑0107

17. Landes, M. et al.Hepatitis B or hepatitis C coinfection in HIV-infected pregnant women in Europe.HIV Medicine.2008, 9(7):526–534. doi: 10.1111/j.1468-1293.2008.00599.x
18. Bigna, J. J. et al.Hepatitis B virus and HIV coinfection among adults residing in Cameroon: A systematic review and meta-analysis of prevalence studies. Infection, Disease and Health.2018,23(3), pp. 170–178. doi: 10.1016/j.idh.
19. Djuidje, N. M. et al.Human immunodeficiency virus and hepatitis B virus (HIV/HBV) co-infection in people living with HIV/AIDs identified in Yaound Central Hospital, Cameroon: Seroprevalence and impact on the disease progression. Journal of AIDS.2017.
20. Lar, P. et al. Prevalence and immune status of HIV/HBV co-infected pregnant women.African Journal of Clinical and Experimental Microbiology.2013,14(3). doi: 10.4314/ajcem.v14i3.1.
21. Thumbiran, N. V. et al.Hepatitis B and HIV co-infection in pregnant women: Indication for routine antenatal hepatitis b virus screening in a high HIV prevalence setting', South African Medical Journal.2014, 104(4): 307–309. doi: 10.7196/SAMJ.7299.
22. Onakewhor, J. U. and Okonofua, F. E.The prevalence of dual human immunodeficiency virus/hepatitis C virus (HIV/HCV) infection in asymptomatic pregnant women in Benin City, Nigeria..African journal of reproductive health.2009,13(2), pp. 97–108. doi: 10.2307/20617116.
23. Ikeako, L. et al. Seroprevalence of human immunodeficiency virus, Hepatitis b, Hepatitis c, syphilis and co-infections among antenatal women in a tertiary institution in south-east Nigeria', Annals of Medical and Health Sciences Research.2014, 4(9):259. doi: 10.4103/2141-9248.141969.
24. Mu, D., Hsa, A. and Kc, A. Rapid screening for co-infection of HIV and HCV in

pregnant women in Benin City , Edo State , Nigeria'. African Health Sciences .2009,9(3): 137-142.

25. Zenebe, Y. et al.Sero-prevalence and risk factors of hepatitis B virus and human immunodeficiency virus infection among pregnant women in Bahir Dar city, Northwest Ethiopia: A cross sectional study', BMC Infectious Diseases.2014, 14(1):1–7. doi: 10.1186/1471-2334-14-118.
26. Desalegn, Z. et al. (2016) 'Hepatitis B and human immunodeficiency virus co-infection among pregnant women in resource-limited high endemic setting, Addis Ababa, Ethiopia: Implications for prevention and control measures.European Journal of Medical Research.2016, BioMed Central, 21(1): 1–7. doi: 10.1186/s40001-016-0211-3.
27. Delelegn Tsegaye., et al.Sero-prevalence and Predictors of Hepatitis B Virus and Hepatitis C Virus Infections among Pregnant Women Attending Antenatal Care in Adjibar Rural Health Center, Northeast Ethiopia. EC Gynaecology .2018,7(11):421-430.
28. Biazin, H. et al.Determining seroprevalencece of Hepatitis B and Cvirus infections and associated risk factors among apparently healthy mothers in Addis Ababab ,Ethiopia. Ethiop Med J, 2019, 129–138.
29. World population review.2019.
30. Ajugwo AO, Mounbegna P, Dunga KE, Eze RI, Erhabor TA(2018). Co-infection of HIV, HBsAg and HCV among Pregnant Women of African Descent. Int J Immunol Immunobio.2018,1(1):103.
31. Ramos JM, Toro C, Reyes F, Amor A, Gutiérrez . Seroprevalence of HIV-1, HBV, HTLV-1 and Treponema pallidum among pregnant women in a rural hospital in Southern Ethiopia. J Clin Virol 2011, 51(1):83–85.
32. Umare, A. et al.Hepatitis B virus infections and associated factors among pregnant

women attending antenatal care clinic at deder hospital, eastern Ethiopia. PLoS ONE.2016,11(11):1–11. doi: 10.1371/journal.pone.0166936.

33. Khan, J. et al. (2014) ‘Seropositivity and Coinfection of Hepatitis B and C among Patients Seeking Hospital Care in Islamabad , Pakistan’. Hindawi Publishing Corporation, BioMed Research International. 2014.doi.org/10.1155/2014/516859.
34. Ephraim, R. et al.Seroprevalence and risk factors of hepatitis B and hepatitis C infections among pregnant women in the asante akim north municipality of the ashanti region, Ghana; a cross sectional study’, African Health Sciences.2015, 15(3):709–713. doi: 10.4314/ahs.v15i3.2.
35. Murad, E. A. et al. (2013) ‘Epidemiology of hepatitis B and hepatitis C virus infections in pregnant women in Sana ’ a , Yemen’. BMC Pregnancy and Childbirth.2013, 13:127 http://www.biomedcentral.com/1471-2393/13/127.
36. Flores, G. L. et al. A cross section study to determine the prevalence of antibodies against HIV infection among hepatitis B and C infected individuals. International Journal of Environmental Research and Public Health.2016, 13(3):7–12. doi: 10.3390/ijerph13030314.
37. Wardani, Y. et al.The Use of Sterile Razors as an Efforts to HIV Transmission Prevention’, International Journal of Public Health Science (IJPHS). 2017,6(4): 337. doi: 10.11591/ijphs.v6i4.10780.
