# Supplementary material for: Co-infection of HIV or HCV among HBsAg positive delivering mothers and its associated factors in governmental hospitals in Addis Ababa, Ethiopia: A cross-sectional study
Source: PLoS One. 2022 Aug 26;17(8):e0273300. doi: 10.1371/journal.pone.0273300 (PMC9417033; doi:10.1371/journal.pone.0273300)
Supplement: Attachment — Submitted filename: Respone to Reviewers.docx [file pone.0273300.s003.docx]

**Rebuttal letter**

**Manuscript Number:** PONE-D-21-13799

**Manuscript title**: Co-infection of HIV or HCV among HBsAg positive delivering mothers and its associated factors in governmental hospitals in Addis Ababa, Ethiopia: a cross-sectional study

**Response to reviewers**

**Dear Edford Sinkala**

Thank you for giving us the opportunity to submit a revised manuscript of **^<<^** Co-infection of HIV or HCV among HBsAg positive delivering mothers and its associated factors in governmental hospitals in Addis Ababa, Ethiopia: a cross-sectional study^>>^ for publication in the journal of PLOS ONE. We appreciate the time and effort that you and the reviewers dedicated to provide feedback on our manuscript and are grateful for the insightful comments on and valuable improvements to our paper. We have incorporated almost all of the suggestions made by the reviewers. Those changes are highlighted within the manuscript. Please see below, in blue, for a point-by-point response to the reviewers’ comments.

**Academic editors and Reviewer’s comment to the authors**

1. There are several parts in the tables where they need to be properly formatted. There are sections of the tables where spacing is off between numbers and percentages.

**Author response:** Thank you for pointing this out. We accepted the comment and we have properly formatted the tables and arranged the spaces between numbers and percentages throughout the manuscript.

2. Fix abbreviations throughout the paper. When referring to Hepatitis B Virus, Hepatitis C Virus, and Human Immunodeficiency Virus, you should spell them out at first use in the paper and put the abbreviation in parentheses right after the first spelled out. Then use the abbreviation throughout the rest of the paper. This could also be done to abbreviate mother-to-child-transmission as MTCT.

**Author response**: we agree with the reviewers and corrected it throughout the paper.

3. Need to consistently use either "HBV-positive" or "HBV positive" throughout the paper. They are both used but one style of writing that needs to be used. This applies to HIV-positive, HCV-positive, and HBsAg-positive as well.

**Author response:** we agree with the reviewers and used HBV positive, HIV positive, HCV positive, and HBsAg positive consistently**.**

4. Be sure to indent the beginning of each paragraph throughout paper.

Author response: we accepted the reviewers comment and corrected it throughout the paper.

5. Check the formatting of reference list and in-text citations to ensure they adhere to journal guidelines.

Author response: we agree and revised it.

6. Be sure to put in-text citations at the end of the sentence, not randomly in the middle.

Author response: we accept the comment and corrected it.

Once more we thank you for the comments. We believe our manuscript is now ready for publishing.

Sincerely,

Mebrihit Arefaine

On behalf of all authors
