# Supplementary material for: Co-infection of HIV or HCV among HBsAg positive delivering mothers and its associated factors in governmental hospitals in Addis Ababa, Ethiopia: A cross-sectional study
Source: PLoS One. 2022 Aug 26;17(8):e0273300. doi: 10.1371/journal.pone.0273300 (PMC9417033; doi:10.1371/journal.pone.0273300)
Supplement: Attachment — Submitted filename: Response to reviewers.docx [file pone.0273300.s004.docx]

**Rebuttal letter**

**Manuscript Number:** PONE-D-21-13799R1

**Manuscript title**: Co-infection of HIV or HCV among HBsAg positive delivering mothers and its associated factors in governmental hospitals in Addis Ababa, Ethiopia: a cross-sectional study

**Response to reviewers**

Dear Natasha McDonald, PhD (Associate Editor)

Thank you for giving us the opportunity to submit a revised manuscript of **^<<^** Co-infection of HIV or HCV among HBsAg positive delivering mothers and its associated factors in governmental hospitals in Addis Ababa, Ethiopia: a cross-sectional study^>>^ for publication in the journal of PLOS ONE. We appreciate the time and effort that you and the reviewers dedicated to provide feedback on our manuscript and are grateful for the insightful comments on and valuable improvements to our paper. We have incorporated all of the suggestions made by the academic editors and reviewers. Those changes are highlighted within the manuscript. Please see below, in blue, for a point-by-point response to the academic editors and reviewers’ comments.

**Academic editors and Reviewer’s comment to the authors**

**Journal requirements**

1. Please review your reference list to ensure that it is complete and correct. If you have cited papers that have been retracted, please include the rationale for doing so in the manuscript text, or remove these references and replace them with relevant current references. Any changes to the reference list should be mentioned in the rebuttal letter that accompanies your revised manuscript. If you need to cite a retracted article, indicate the article’s retracted status in the References list and also include a citation and full reference for the retraction notice.

**Author response:** Thank you for pointing this out. We accepted the comment, and we have reviewed the reference list. We found that references number 27 and 29 were retracted papers and replaced with other current references. References number 3 and 24 were wrongly done and corrected in the revised manuscript.

2. The authors should provide key words at the end of the abstract (Reviewer 3)

**Author response:** Based on the submission guideline format of PLOS ONE journal, keywords should not be included at the end of the abstract. But, they incorporate during online submission. That is why we cannot provide keywords at the end of the abstract.

3. The authors should include the prevalence of cases with triplex infections.

**Author response:** we accepted the reviewers’ comment and included in the manuscript.

4. **REFERENCES**

Some references were wrongly done. Example, reference 24 should be: Duru MU, Aluyi HS, Anukam KC. Rapid screening for co-infection of HIV and HCV in pregnant women in Benin City, Edo State, Nigeria. Afr Health Sci. 2009 Sep;9(3):137-42. PMID: 20589140; PMCID: PMC2887022.

**Author response**: we accept the comment and corrected it.

Once more we thank you for the comments. We believe our manuscript is now ready for publishing.

Sincerely,

Mebrihit Arefaine

On behalf of all authors
